# Generation and phenotypic characterization of *Pde1a* mutant mice

**DOI:** 10.1371/journal.pone.0181087

**Published:** 2017-07-27

**Authors:** Xiaofang Wang, Satsuki Yamada, Wells B. LaRiviere, Hong Ye, Jason L. Bakeberg, María V. Irazabal, Fouad T. Chebib, Jan van Deursen, Peter C. Harris, Caroline R. Sussman, Atta Behfar, Christopher J. Ward, Vicente E. Torres

**Affiliations:** 1 Division of Nephrology and Hypertension, Mayo Clinic, Rochester, Minnesota, United States of America; 2 Department of Cardiovascular Diseases, Mayo Clinic, Rochester, Minnesota, United States of America; 3 Division of Nephrology and Hypertension, University of Kansas Medical Center, Kansas City, Kansas, United States of America; 4 Department of Biochemistry and Molecular Biology, Mayo Clinic, Rochester, Minnesota, United States of America; Universita degli Studi di Bari Aldo Moro, ITALY

## Abstract

It has been proposed that a reduction in intracellular calcium causes an increase in intracellular cAMP and PKA activity through stimulation of calcium inhibitable adenylyl cyclase 6 and inhibition of phosphodiesterase 1 (PDE1), the main enzymes generating and degrading cAMP in the distal nephron and collecting duct, thus contributing to the development and progression of autosomal dominant polycystic kidney disease (ADPKD). In zebrafish *pde1*a depletion aggravates and overexpression ameliorates the cystic phenotype. To study the role of PDE1A in a mammalian system, we used a TALEN pair to *Pde1a* exon 7, targeting the histidine-aspartic acid dipeptide involved in ligating the active site Zn^++^ ion to generate two *Pde1a* null mouse lines. *Pde1a* mutants had a mild renal cystic disease and a urine concentrating defect (associated with upregulation of PDE4 activity and decreased protein kinase A dependent phosphorylation of aquaporin-2) on a wild-type genetic background and aggravated renal cystic disease on a *Pkd2*^WS25/-^ background. *Pde1a* mutants additionally had lower aortic blood pressure and increased left ventricular (LV) ejection fraction, without a change in LV mass index, consistent with the high aortic and low cardiac expression of *Pde1a* in wild-type mice. These results support an important role of PDE1A in the renal pathogenesis of ADPKD and in the regulation of blood pressure.

## Introduction

Autosomal dominant polycystic kidney disease (ADPKD) is the fourth leading cause of end-stage kidney disease. It is caused by mutations in *PKD1* or *PKD2* encoding polycystin 1 and polycystin 2 [1, 2]. Substantial evidence supports the hypothesis that disruption of polycystin function results in dysregulation of intracellular calcium dynamics and upregulation of 3',5'-cyclic adenosine monophosphate (cAMP) and protein kinase A (PKA) signaling [3–5]. The identification of cAMP and PKA signaling as a therapeutic target [6–9] has led to clinical trials of vasopressin V2 receptor (V2R) antagonists and somatostatin analogs [10, 11] and to the recent approval of the V2R antagonist, tolvaptan, for the treatment of ADPKD with rapidly progressive renal disease in Japan, Canada, the European Union, Switzerland and South Korea.

Further understanding of the mechanisms responsible for the increased cAMP signaling in PKD may provide additional therapeutic opportunities. It has been proposed that the increase in cAMP signaling is, in part, a direct consequence of a reduction in intracellular calcium homeostasis through the inhibition of phosphodiesterase (PDE)-1, the only PDE activated by calcium [6]. The PDE1 family consists of three isoforms encoded by three distinct genes, *PDE1A*, *PDE1B* and *PDE1*C. We have shown that *pde1a* interference using splice- and translation-blocking morpholinos causes pronephric cysts, hydrocephalus, and body curvature in wild-type zebrafish embryos and aggravates the cystic phenotype in *pkd2* morphants, while human *PDE1A* RNA partially rescues the *pde1a* and *pkd2* morphant phenotypes [12]. To study the role of PDE1A in a mammalian system we created Pde1a null mouse lines using TALENs.

## Methods

The Mayo Clinic Institutional Animal Care and Utilization Committee approved all experimental protocols for the work described within this report.

### Targeted disruption of *Pde1a*

**Fig 1:**
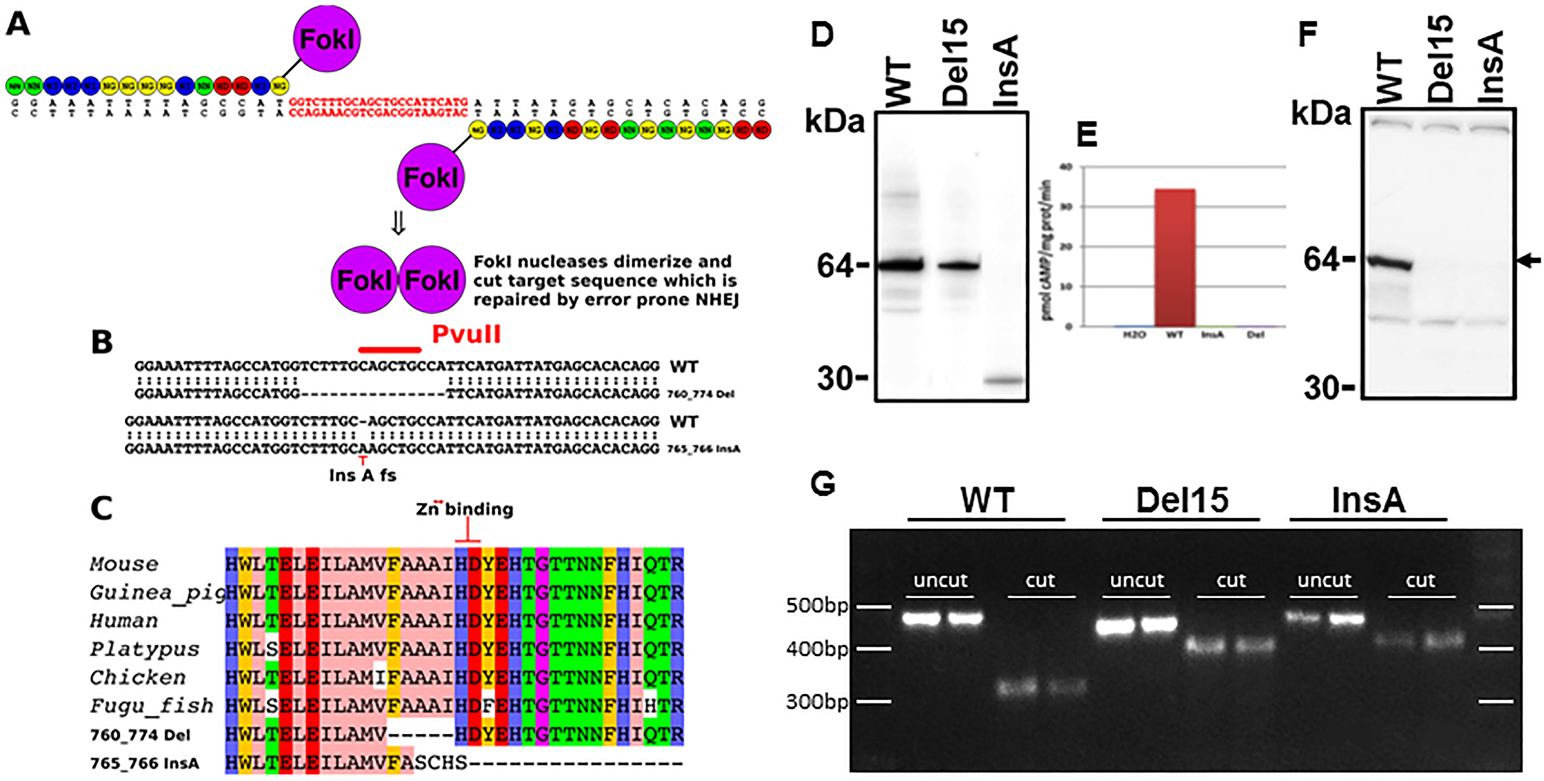
A) Left and right TALENs designed to disrupt exon 7 of mPde1a (active site). B) Recovered mutations include an in-frame 15bp deletion and insertion of a single A. C) Effect on mPde1a protein, the 15bp in frame deletion (760_774) removes amino acids FAAAI and the insertion of an A (765_766) truncates mPde1a after four out of frame amino acids. D) Western blot of N-terminal V5-tagged wild-type and mutant mPde1a expressed in *Xenopus* oocytes detected with V5 antibody. E) PDE1 activity (Ca^2+^/calmodulin dependent hydrolysis of cAMP) in *Xenopus* oocytes injected with water, wild-type RNA or mutated RNAs. F) Western blot of kidney lysates showing a 64 kDa protein in wild-type mice (arrow) detected with a rabbit polyclonal antibody raised against recombinant human PDE1A (360 C-terminal amino acids); this band was markedly reduced in *Pde1a*^Del15^ and absent in *Pde1a*^InsA^ homozygous mice. G) Reverse transcription of kidney RNA, PCR amplification and PvuII-HF restriction endonuclease digestion of a 485 bp PCR product, showing a 327 bp fragment in the wild-type and a 420 bp fragment in the mutants (smaller digest fragments are not resolved).

We used a TALEN pair to *Pde1a* exon 7 (NM_001159582.1 mouse chromosome 2). This exon was selected because it is the second exon in the catalytic domain and contains a histidine-aspartic acid dipeptide involved in the coordination of a Zn^++^ ion required for catalytic activity [13] (Fig 1A). We injected a total of 100 C57BL6/J oocytes, recovered 40 pups of which at least 5 males and 3 females harbored a mutation.

### Genotyping and breeding of *Pde1a* and *Pkd2* mutant mice

*Pde1a* (Fig A in S1 File) and *Pkd2* genotyping by PCR and Southern blot are described in the Supplemental material. *Pde1*a^Del15/Del15^, *Pkd2*^+/-^ and *Pkd2*^WS25/WS25^ mice were crossed to generate *Pde1a*^Del15/Del15^;*Pkd2*^-/WS25^ and *Pde1a*^*+/+*^;*Pkd2*^-/WS25^ mice.

### Phenotypic characterization of *Pde1a* mutant mice

Litter sizes, blood and urine biochemistries and magnetic resonance imaging (MRI) of the abdomen and/or heart were obtained at 6 and 12 months in *Pde1a* mutant and wild-type mice. Urinary concentrating ability and the capacity to excrete a water load were tested at 12 months of age. Echocardiogram, aortic blood pressure and histology of *Pde1a*^Del15/Del15^ and *Pde1a*^InsA/InsA^ were compared to those of sex and age matched wild-type controls.

### Treatment with desmopressin

Desmopressin (30 ng/100 g/hour) or saline vehicle was administered subcutaneously via osmotic minipumps (Alzet 1004 replaced every 3 wk) to wild-type and *Pde1a*^InsA/InsA^ mice, or to wild-type;*Pkd2*^-/WS25^ and *Pde1a*^Del15/Ddel15^;*Pkd2*^-/WS25^ mice between 4 and 16 weeks of age.

### Abdominal and cardiac MRI

Ultra high field (UHF) abdominal and cardiac MRI images were acquired as described in the supplemental material (https://www.jove.com/video/52757/use-ultra-high-field-mri-small-rodent-models-polycystic-kidney).

### Blood collections and tissue harvesting

Blood was obtained by cardiac puncture under ketamine (60mg/kg i.p.) and xylazine (10mg/kg i.p.) anesthesia. The right kidney and part of the liver were placed into preweighed vials containing 10% formaldehyde in phosphate buffer. The left kidney was immediately frozen in liquid nitrogen. Hearts were cut transversally and the apex was frozen immediately in liquid nitrogen while the rest was placed into vials containing 10% formaldehyde.

### PDE activities

PDE activities in the kidneys and heart were measured as described in the Supplemental Material. Specific activities were expressed as picomoles of cAMP hydrolyzed per minute/mg of protein.

### cAMP and cGMP content

The cAMP and cGMP were assessed by enzyme-linked immunosorbent assay (Enzo Life Sciences, Farmingdale, NY). Results were expressed in pmol/mg of protein.

### Western blots

Immunoblotting of kidney and heart lysates or cytosol was performed as described in the Supplemental Material. Antibodies used were: PDE1A (12442-2-AP, Proteintech, Rosemount IL); PDE1B (ab14600, Abcam, Cambridge, MA); PDE1C (sc67323, Santa Cruz, CA); pSer269-AQP2 (ab110418, Abcam, MA). The membrane was stained using swift membrane stain kit and total protein stain was used as loading control [14].

### RT-PCR of tissue RNA

1μg of total RNA was reverse transcribed using SuperScript First-Strand Synthesis System (Invitrogen) in a total volume of 20μl at 37^°^C for 1hour. The PCR reactions were performed with 200nM mouse Pde1a specific primers: Forward: 5’-ATGCAGCTGACGTCACTCAA, Reverse: 5’- AGGGCCATGGTCCATCTGTA for 30 cycles at 95^°^C for 40s, 60^°^C for 1min and 72^°^C for 1min. 10μl of the PCR products were digested with 10μl of a digest master mix using 0.3μl of PvuII-HF per reaction and digested overnight. The uncut PCR product is 484 bp. PvuII cut the PCR product to generate fragments of 327+93+58+6 bp in wild-type and 420+58+6 bp in *PDE1a*^Del15^ and *PDE1a*^InsA^.

### Histomorphometric and immunohistochemical analyses

These were performed as described in the Supplemental material. Antibodies used were against lysozyme (ab108508, Abcam, Cambridge, MA), Tamm-Horsfall protein (THP, sc20631, Santa Cruz, CA), aquaporin-2 (AQP2, sc9882, Santa Cruz, CA), epithelial membrane antigen (EMA, MA5-11202, Thermo Scientific, Rockford, IL), and proliferating cell nuclear antigen (PCNA, sc-56, Santa Cruz, CA).

### Statistical analysis

Data are expressed as means ± SD. One-way analysis of variance (ANOVA) with post-hoc Tukey test is used for comparisons between groups. The Student’s *t*-test was used for comparisons between two groups.

## Results

### *Pde1a* mutants

Of eight pups carrying *Pde1a* mutations, we focused on an in frame deletion of 15bp, c.760-774del p.Phe254-Ile258del deleting amino acids FAAAI in the active site of mPDE1A (*Pde1a*^Del15^) and an A insertion in the same region c.765-766insA p.Ala255fs5X which caused a frame shifting mutation that terminated the open reading frame after 4 out of frame amino acids (*Pde1a*^InsA^) (Fig 1B and 1C). V5-tagged wild-type, *Pde1a*^Del15^ and *Pde1a*^InsA^ RNAs were injected into Xenopus oocytes. Western blotting with a V5 antibody showed a 64 kDa protein in the oocytes injected with the wild-type or *Pde1a*^Del15^ RNA and a truncated 30 kDa protein in oocytes injected with the *Pde1a*^InsA^ RNA (Fig 1D). In contrast to wild-type RNA, mutated RNAs failed to generate calcium/calmodulin dependent enzymatic activity when injected into Xenopus oocytes (Fig 1E). Western blot analysis of kidney lysates from wild-type mice with a PDE1A polyclonal antibody showed a 64 kDa protein, which was markedly reduced in *Pde1a*^Del15^ and absent in *Pde1a*^InsA^ mice (Fig 1F). Reverse transcription of kidney RNA, PCR amplification and PvuII-HF restriction endonuclease digestion of the 485 bp PCR product showed 6, 59, 93, and 327 bp fragments in the wild-type 6, 59, and 420 bp fragments in the mutants, confirming the genotyping results (Fig 1G).

### Characteristics of *Pde1a* knockout mice

The Pde1a alleles were produced on an inbred B57BL/6J background, identical to the background used to inbreed our PKD models. Male and female *Pde1a* mutant mice were fertile. Litter sizes, general appearance and pre-weaning or post-weaning growth of homozygous or heterozygous *Pde1a*^Del15^ or *Pde1a*^InsA^ were not different from wild-type mice. At 12 months of age, kidney to body weight ratios were not different between *Pde1a*^Del15/Del15^ and *Pde1a*^InsA/InsA^ mice but both were significantly higher compared to the wild-type mice, whereas body weights, weights of other organs, and blood chemistries were similar (Table 1). Subsequently, both the homozygous *Pde1a*^Del15^ and *Pde1a*^InsA^ mice were considered as null models.

**Table 1:** Body and organ weights and laboratory parameters at 12 months of age.

	Wild-type (10 M, 9 F)	*Pde1a*^InsA/InsA^ (9 M, 9 F)	*Pde1a*^Del15/Del15^ (12 M, 19 F)	*Pde1a*^InsA/InsA or Del15/Del15^ (21 M, 28 F)
Body weight, g	29.4±5.3	29.2±3.6	28.7±3.7	28.9±3.7
Kidney weight, g	0.42±0.08	0.45±0.06	0.47±0.09	0.46±0.08
Kidney weight, % BW	1.44±0.11	1.54±0.13*	1.63±0.26^‡^	1.60±0.22^‡^
Liver weight, g	1.48±0.31	1.32±0.23	1.40±0.30	1.37±0.28
Liver weight, % BW	5.03±0.57	4.56±0.79	4.88**±0.72**	4.76±0.76
Heart weight, g	0.18±0.05	0.20±0.03	0.20±0.04	0.20±0.12
Heart weight, % BW	0.63±0.10	0.68±0.12	0.69±0.11	0.69±0.12
Spleen weight, g	0.09±0.05	0.11±0.04	0.11±0.04	0.11±0.04
Spleen weight, % BW	0.34±0.13	0.39±0.19	0.38±0.14	0.38±0.16
Lung weight, g	0.22±.04	0.23±0.04	0.25±0.04	0.24±0.04
Lung weight, % BW	0.78±0.18	0.81±0.20	0.88±0.18	0.86±0.19
Serum sodium, mEq/L	152.9±4.5	149.1±3.8	151.3±4.4	150.5±4.3
Serum potassium, mEq/L	7.1±0.9	7.0±1.1	7.3±1.1	7.2±1.1
Serum glucose, mg/dL	174±27	207±32	171±28	185±34
Serum creatinine, mg/dL	0.47±0.24	0.49±0.15	0.53±0.17	0.51±0.61

vs wild-type

*p<0.05

‡p<0.001.

BW: Body weight

### Activity and expression of PDE1, PDE3 and PDE4 families and cAMP levels in kidneys and hearts from *Pde1a*^Del15^ and *Pde1a*^InsA^ homozygotes compared to wild-type mice

**Fig 2:**
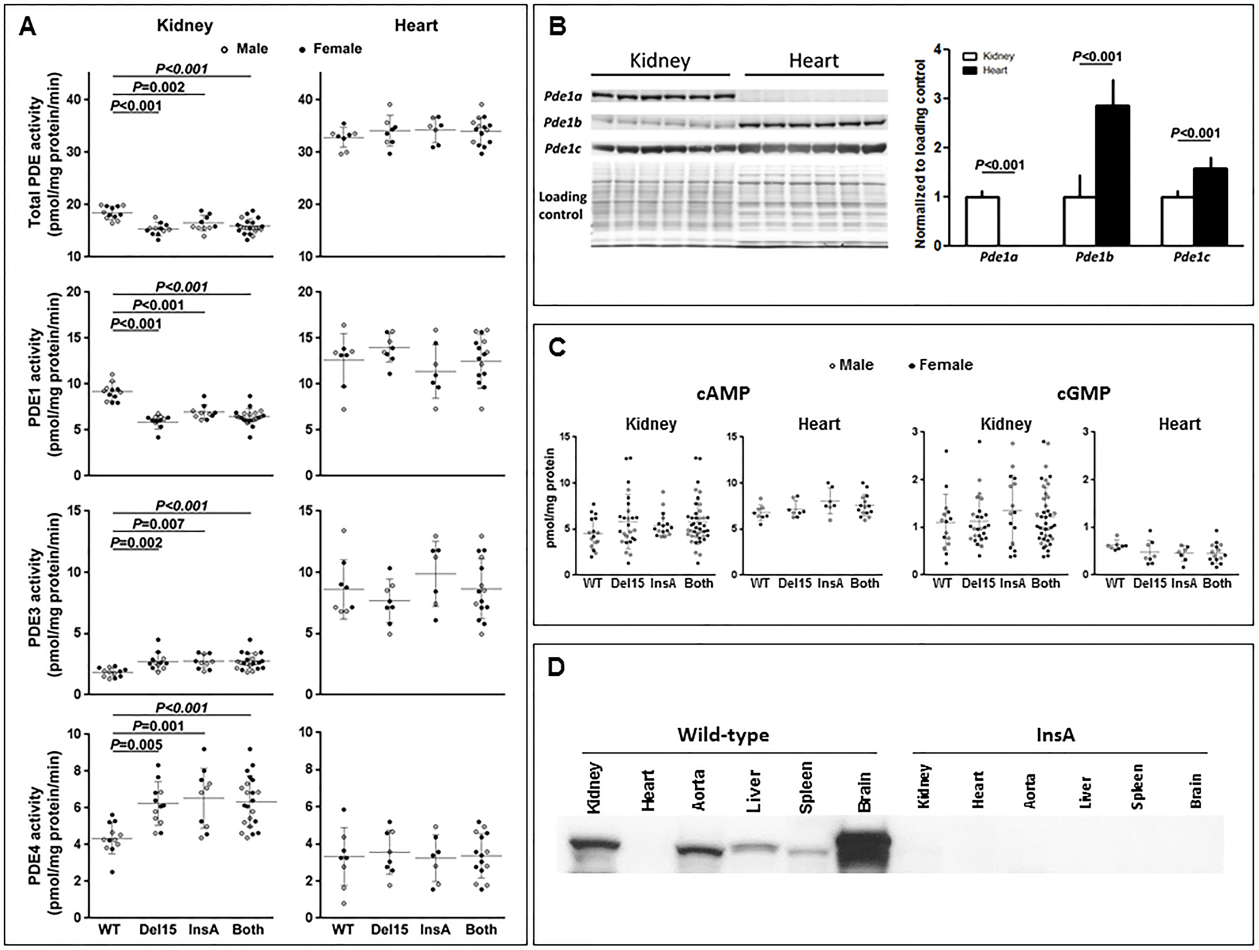
A) Total PDE, PDE1, PDE3 and PDE4 activities in kidney and heart lysates from wild-type mice and from Pde1aDel15 and Pde1aInsA homozygous mice or results of both combined. B) Western blots of kidney and heart lysates from six wild-type mice detected with PDE1A, PDE1B and PDE1C antibodies; total protein stain with Coomassie Blue is used as a loading control. C) Cyclic AMP and cGMP levels in kidney and heart lysates from wild-type mice and from *Pde1a*^Del15^ and *Pde1a*^InsA^ homozygous mice or results of both combined. D) Western blot of kidney, heart, aorta, liver, spleen and brain lysates from wild-type and *Pde1a*^InsA^ homozygous mice detected with a PDE1A antibody; a weak cardiac band can be seen in wild-type with longer exposure (data not shown). One-way ANOVA with post-hoc Tukey test was used for the statistical analysis.

In kidney tissue total PDE and PDE1 activities were reduced, whereas PDE3 and PDE4 activities were increased in both, *Pde1a*^Del15/Del15^ mice, as previously reported in younger animals[15], and in *Pde1a*^InsA/InsA^ mice compared to wild-type controls (Fig 2A). Total PDE, PDE1 and PDE3 activities were higher in the hearts than in the kidneys. In contrast to the kidneys, cardiac PDE1 activities were not lower and PDE3 and PDE4 activities were not higher in either model compared to wild-type mice. Expression of PDE1A protein was lower and expressions of PDE1B and PDE1C proteins were higher in hearts compared to kidneys of wild-type mice (Fig 2B). Renal cAMP levels tended to be higher in the kidneys but not in the hearts of *Pde1a* mutants compared to control animals (Fig 2C). Renal and cardiac cGMP levels in the Pde1a mutant and wild-type mice were not different (Fig 2C). PDE1A protein was expressed at higher levels in the aorta compared to the heart, with the highest level of expression being in the brain (Fig 2D).

### *Pde1a* null mice developed mild renal cystic disease

**Fig 3:**
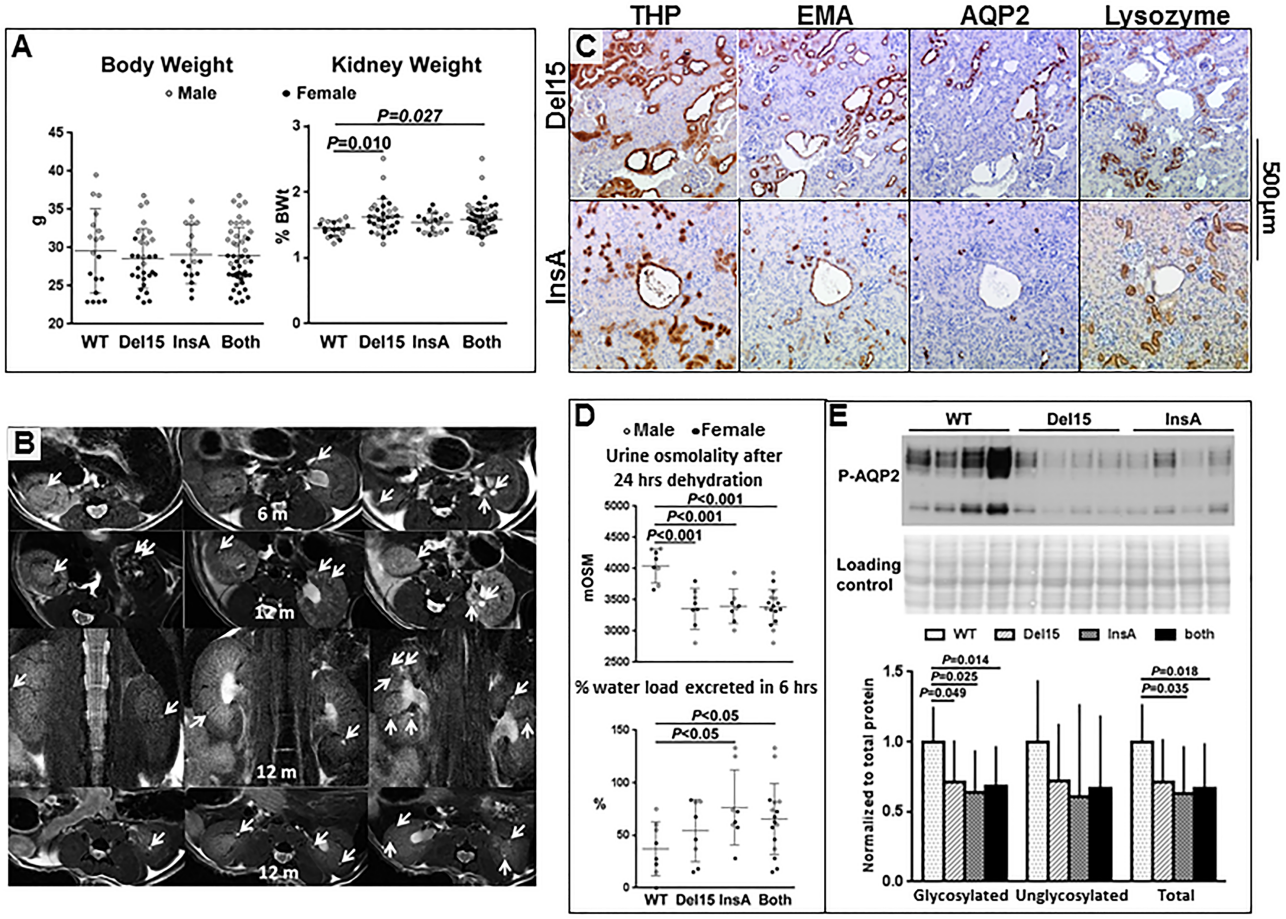
A) Body weight and kidney weight as percent of body weight (%KW/BW) in wild-type mice and in *Pde1a*^Del15^ and *Pde1a*^InsA^ homozygous mice or data from both combined. B) Axial and coronal MR images of *Pde1a* mutant mice at 6 and 12 months of age showing small renal cysts (arrows; this panel is also shown as a larger figure in Fig B in S1 File). C) Kidney sections from *Pde1a*^Del15^ and *Pde1a*^InsA^ homozygous mice showing multiple small cysts staining positively for Tamm-Horsfall protein and epithelial membrane antigen, less consistently for aquaporin-2, and not staining for lysozyme. D) Reduced urine osmolality after 24 hours of dehydration, faster excretion of an acute water load, and E) reduced expression of glycosylated (upper band) and unglycosylated (lower band) pSer269-AQP2 in *Pde1a*^Del15^ and *Pde1a*^InsA^ homozygotes compared to wild-type mice. One-way ANOVA with post-hoc Tukey test was used for the statistical analysis.

**Fig 4:**
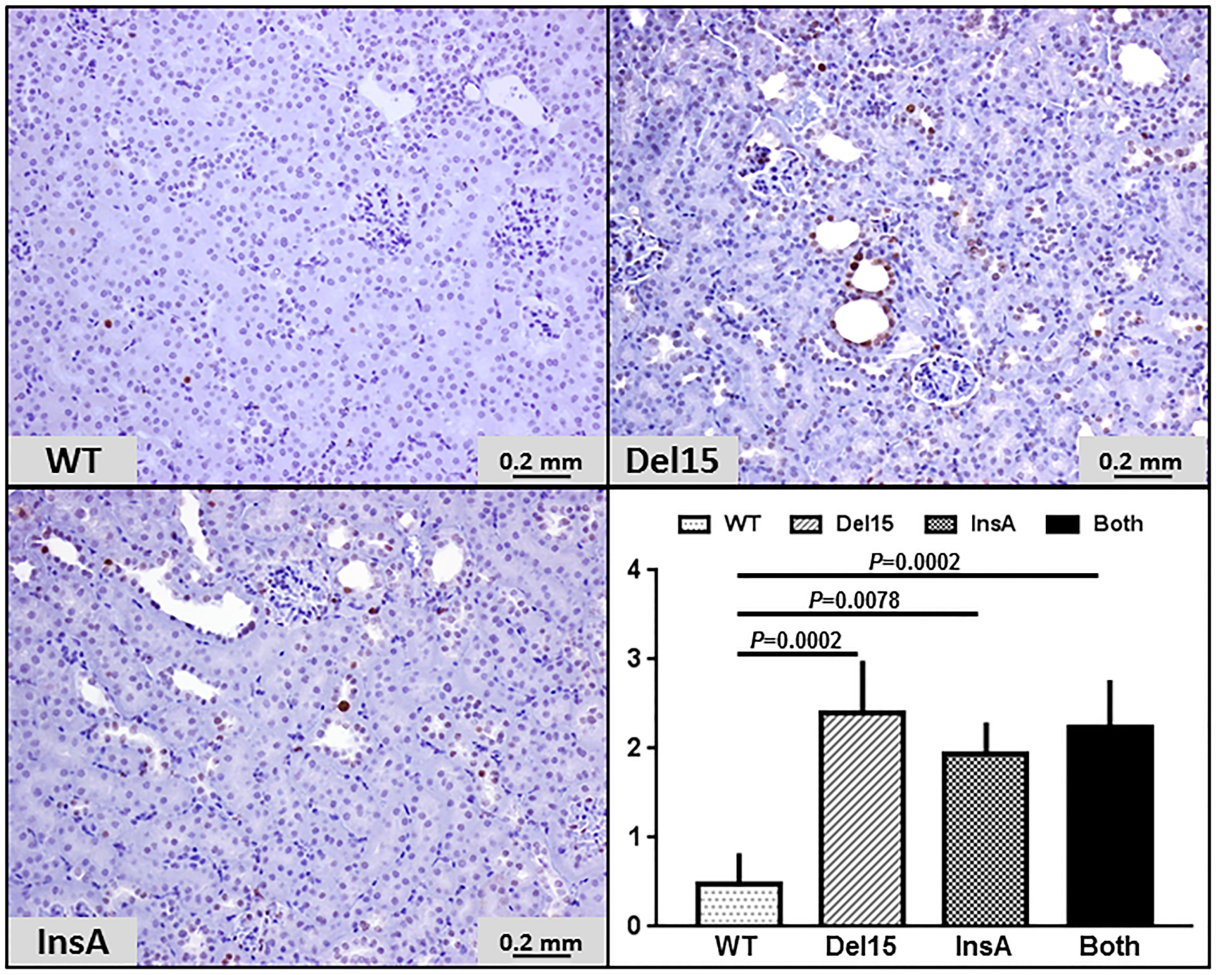
PCNA immunostaining of kidney sections (x 200) in wild-type mice (n = 4) and in *Pde1a*^Del15^ (n = 5) or *Pde1a*^InsA^ (n = 3) homozygous mice, and both combined (n = 8). Proliferative indices were significantly higher in the *Pde1a* mutants compared to the wild type mice. One-way ANOVA with post-hoc Tukey test was used for the statistical analysis.

At sacrifice the Kidney to body weight ratios of the *Pde1a*^InsA/InsA^ and *Pde1a*^Del15/Del15^ homozygous mice were higher than those of wild-type mice (Table 1, Fig 3A). MR scans of the kidneys were obtained at 6 and 12 months of age. At these ages small renal cysts were observed in 9 of 19 (47%, 8 *Pde1a*^Del15/Del15^ and 11 *Pde1a*^InsA/InsA^ mice) and 17 of 27 (63%, 20 *Pde1a*^Del15/Del15^ and 7 *Pde1a*^InsA/InsA^ mice) *Pde1a* mutant compared to 1 of 12 (13%, P = 0.046) and 1 of 10 (10%, P = 0.008) wild-type mice, respectively (Fig 3B and Fig B in S1 File). Histological examination of the kidneys at 12 months of age confirmed the presence of small cortical and medullary cysts and tubular dilatation in 26 of 44 (59%, 26 *Pde1a*^Del15/Del15^ and 18 *Pde1a*^InsA/InsA^ mice) *Pde1a* mutant compared to 3/18 (17%, P = 0.004) wild-type mice (P<0.001). Only one or two small cysts confined to the inner medulla were observed in the three wild-type mice. MR and tissue sections showing small cysts in 12 month old wild-type mice are shown in Fig C in S1 File. Most dilated tubules and microscopic cysts stained positive with Tamm-Horsfall protein (THP, a marker for the thick ascending limb of Henle) and/or epidermal membrane antigen (EMA, a marker for the distal nephron and collecting ducts), less often with aquaporin-2 (AQP-2, a marker for collecting duct principal cells), and were consistently negative for lysozyme (a proximal tubule marker; Fig 3C). Many cells in dilated tubules and microscopic cysts stained positive for PCNA (Fig 4).

### *Pde1a* knockout mice had impaired urine concentrating capacity

The pool of cAMP generated in response to vasopressin is mainly hydrolyzed by PDE1 and the accumulation of cAMP in response to vasopressin is markedly increased when intracellular calcium is reduced, mainly due to lower PDE1 activity [16, 17]. We therefore anticipated that *Pde1a* knockout mice would have an enhanced urine concentrating capacity and impaired diluting capacity. Contrary to this expectation, we found that at 12 months of age these mice had a mild urine concentration defect after 24 hrs dehydration (3375±312 and 3394±273 compared to 4057±255 mOsm/L in wild-type, P<0.01, Fig 3D) and were able to excrete a water load faster (54±29 and 77±35 compared to 37±25% of 2 ml ip over 6 hrs in wild-type mice, P<0.01, Fig 3D). Since PDE4D controls a cAMP pool that regulates the expression, phosphorylation and translocation of AQP2 to the apical membrane of the collecting duct principal cells [18] and constitutive activation of PDE4 was found to be responsible for a mouse model of nephrogenic diabetes insipidus [19], we wondered whether the increased activity of PDE4 in the kidneys of the *Pde1a* mutants could be responsible for the mild concentration defect observed in these mice. Consistent with this, the expression of pSer269-AQP2 was decreased in the kidneys of *Pde1a*^Del15^ and *Pde1a*^InsA^ homozygous mice compared to those of wild-type controls (Fig 3D).

### Effect of desmopressin administration on the renal phenotype of *Pde1a*^InsA/InsA^ mice

**Fig 5:**
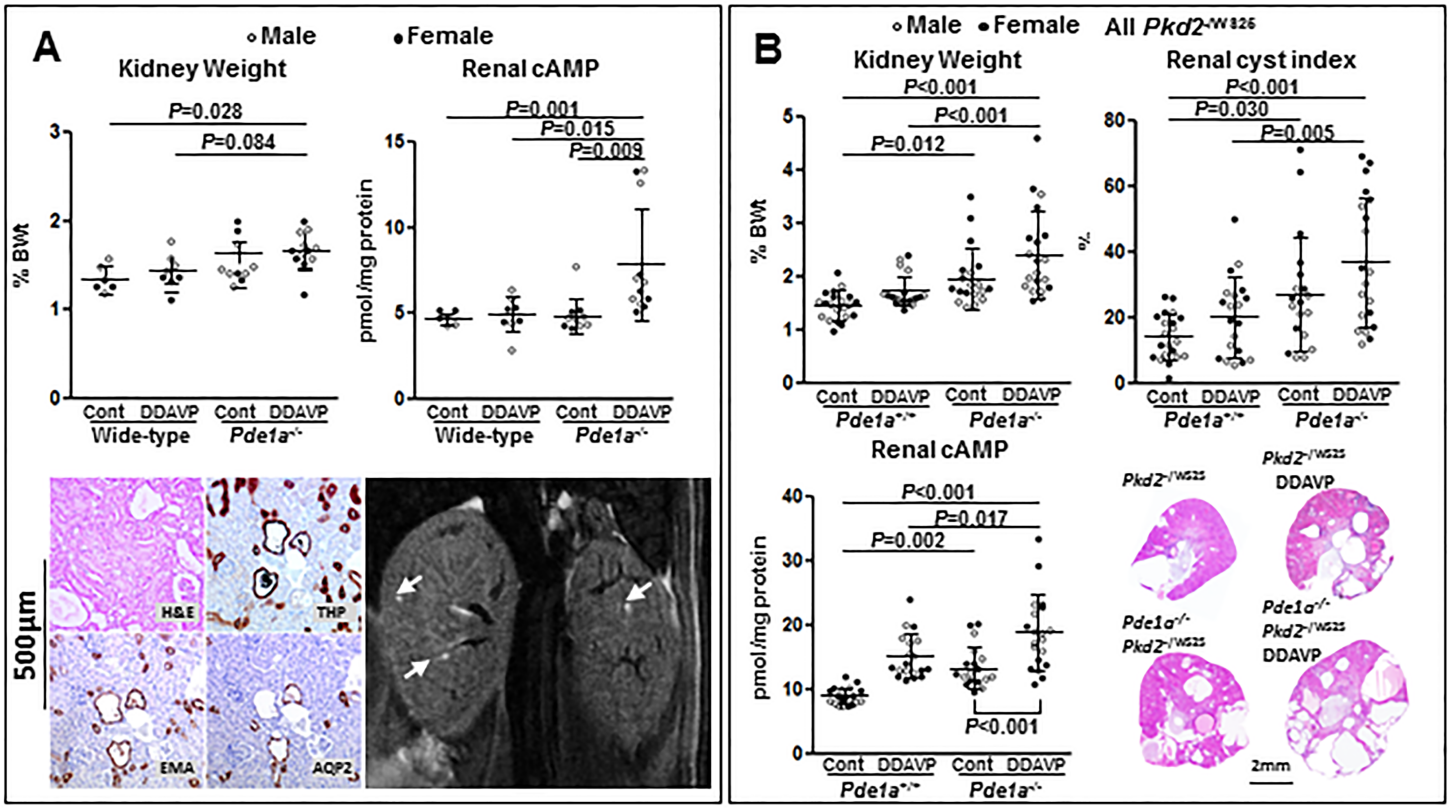
A) Upper panels: Kidney weights as percent of body weights (%KW/BW) and renal cAMP levels in untreated or desmopressin treated wild-type and *Pde1a*^InsA^ homozygous mice (upper panels); One-way ANOVA with post-hoc Tukey test was used for the statistical analysis; using a two-way ANOVA, both *Pde1a* null genotype P<0.001) and desmopressin treatment (P = 0.004) were associated with higher kidney to body weight ratios. Lower panels: Kidney sections stained with hematoxylin-eosin or immunostained with Tamm-Horsfall protein, epithelial membrane antigen or aquaporin-2 antibodies, and coronal MR image showing small cysts (arrows) in desmopressin treated *Pde1a*^InsA^ homozygous mice. B) %KW/BW, kidney cystic indices, renal cAMP levels, and kidney sections stained with hematoxylin-eosin in untreated or desmopressin treated *Pkd2*^-/WS25^ and *Pkd2*^-/WS25^;*Pde1a*^Del15/Del15^ mice. One-way ANOVA with post-hoc Tukey test was used for the statistical analysis.

The administration of desmopressin enhances the development of renal cystic disease in PCK rats and in *Pkd1*^RC/RC^ and *Pkd2*^WS25/-^ mice [8, 20]. To determine whether it could induce the development of renal cystic disease in *Pde1a* mutant mice, desmopressin (DDAVP, 30 ng/100 g BW/hr subcutaneously) or saline was administered to *Pde1a*^InsA^ homozygous and wild-type mice (5 male and 5 female mice per genotype and treatment) between 4 and 16 weeks of age. At 16 weeks of age the kidney to body weight ratios of desmopressin-treated *Pde1a* mutant mice were higher than those of saline-treated wild-type mice (Fig 5A). Two-way ANOVA showed that both *Pde1a* null genotype P<0.001) and desmopressin treatment (P = 0.004) were associated with higher kidney to body weight ratios. Additionally, cAMP levels were higher in desmopressin-treated *Pde1a* mutant mice compared to control mutant and both wild-type groups. Prior to sacrifice, MRI of the kidneys were obtained in five *Pde1a*^InsA^ and four wild-type mice treated with desmopressin; three of the mutant and none of the wild-type mice had renal cysts (2–3 per animal, Fig 5A). Histological examination confirmed the presence of microscopic cysts staining positive for THP, EMA and less consistently AQP2 (Fig 5A).

### The development of polycystic kidney disease in *Pkd2*^-/WS25^ mice was enhanced on a *Pde1a* null genetic background and aggravated by the administration of desmopressin

We previously reported that the development of PKD in *Pkd2*^-/WS25^ mice is aggravated on a *Pde1a* or a *Pde3a* null background [15]. We also suggested that the lower susceptibility to PKD and enhanced disease associated with desmopressin treatment, when comparing mice to rats, may be due to higher PDE activities in mouse compared to rat kidneys [20, 21]. Indeed we found that administration of desmopressin aggravated the renal cystic disease of *Pkd2*^-/WS25^ mice on a *Pde3a* null background compared desmopressin-treated *Pkd2*^-/WS25^ mice on a wild-type background [15]. To determine whether the cystogenic effects of desmopressin would also be enhanced on a *Pde1a* null background, we administered desmopressin (30 ng/100 g BW per hour subcutaneously) or saline to *Pkd2*^-/WS25^;*Pde1a*^Del15/Del15^ and *Pkd2*^-/WS25^ mice. Kidney to body weight ratios and cystic indices were significantly higher in desmopressin-treated *Pkd2*^-/WS25^ mice on a *Pde1*a null background compared to saline- or desmopressin-treated *Pkd2*^-/WS25^ mice on a wild-type background (Fig 5B). Kidney to body weight ratios KWand cystic indices were numerically higher in desmopressin-treated compared to saline-treated *Pkd2*^-/WS25^ mice on a *Pde1a* null background, without reaching statistical significance (P = 0.054 and 0.20, respectively).

### *Pde1a* knockout mice have a cardiovascular phenotype

**Fig 6:**
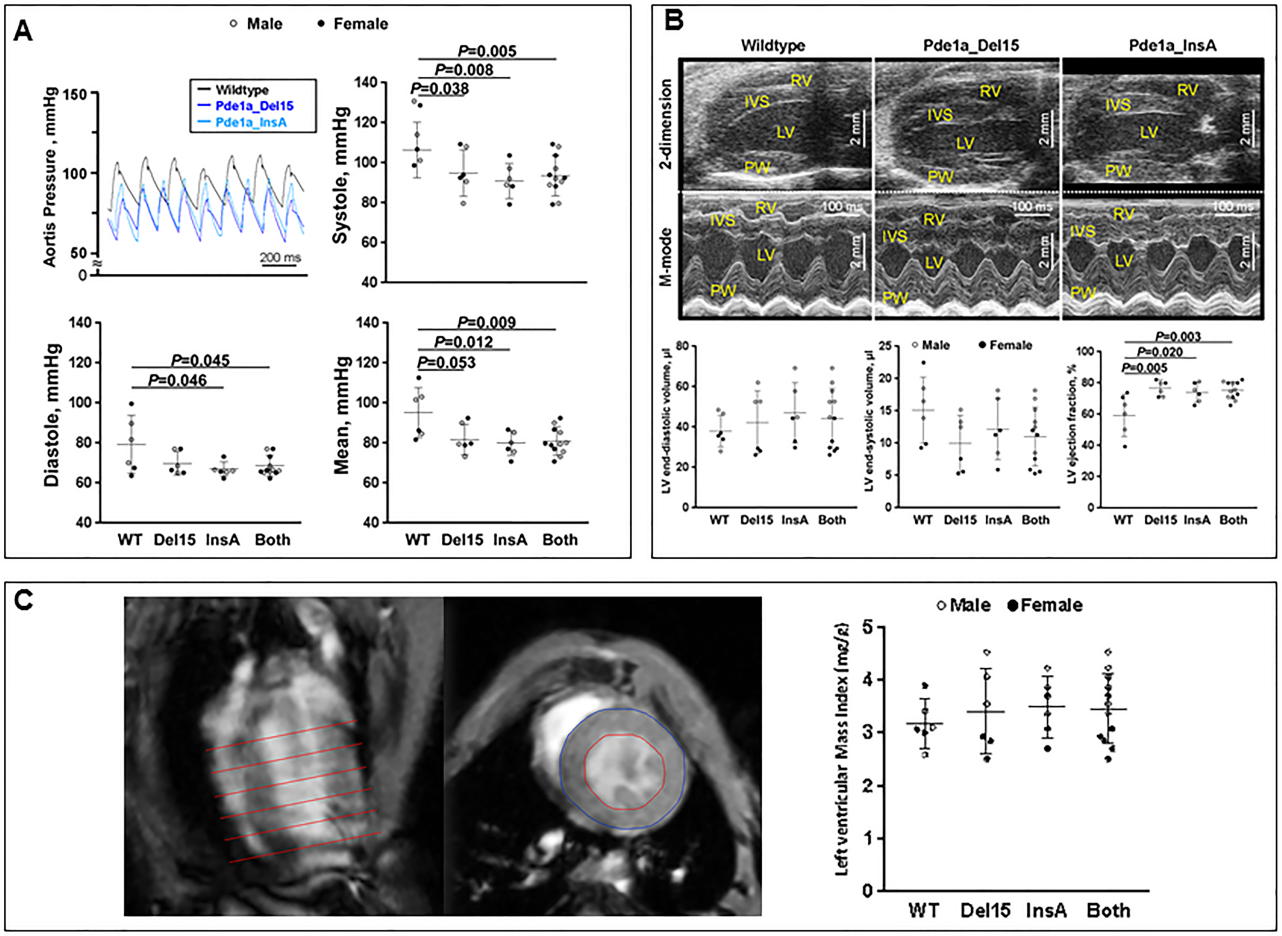
A) Aortic blood pressures were lower in *Pde1a*^Del15^ and *Pde1a*^InsA^ homozygous mice compared to wild-type mice. B) Echocardiographic studies showing increased LV ejection fractions in *Pde1a*^Del15^ and *Pde1a*^InsA^ homozygous mice compared to wild-types; RV: Right ventricle; LV: Left ventricle; IVS: Interventricular septum; PW: Posterior wall C) Measurements of LV mass index using MRI show no significant difference between wild-type and *Pde1a* mutant mice. One-way ANOVA with post-hoc Tukey test was used for the statistical analysis.

Because of the association of various cardiac phenotypes with PKD [22, 23] and the finding that PDE1 accounts for a major component of PDE activity in the heart, we tested whether inactivation of *Pde1a* results in a cardiac or cardiovascular phenotype. We found that both *Pde1a*^Del15^ and *Pde1a*^InsA^ mice had lower aortic blood pressures (Fig 6A) and higher LV ejection fractions on echocardiography (Fig 6B) compared to wild-type mice. However, there was no difference in LV mass index measured by MRI between *Pde1a* wild-type and mutant mice (Fig 6C).

## Discussion

A substantial body of evidence indicates that cAMP and PKA signaling play a central role in PKD [3–5]. It has been proposed that a reduction in intracellular calcium causes the upregulation of cAMP and PKA through stimulation of calcium inhibitable adenylyl cyclase 6 and inhibition of PDE1, the main enzymes generating and degrading cAMP in the distal nephron and collecting duct [6]. Consistent with this hypothesis, a collecting duct-specific knockout of adenylyl cyclase 6 affords protection in a *Pkd1* mouse model [24]. Since capacity for hydrolysis of cyclic nucleotides by PDEs far exceeds that for synthesis by adenylyl cyclases [25], modulation of PDE activity, particularly PDE1 activity, may be crucial in PKD.

PDE1 is one of eleven mammalian PDE families (PDE1-PDE11) with twenty-two genes and close to 100 distinct isoenzyme variants generated through the use of alternate promoter initiation sites and/or alternate splicing [26, 27], PDE family members may exclusively hydrolyze cAMP or cGMP, or they may hydrolyze both. PDE activities are regulated by phosphorylation/dephosphorylation (e.g. PDE4 activation by PKA phosphorylation), binding of cGMP (e.g. PDE3 inhibition by cGMP) or cAMP, binding of calcium/calmodulin (PDE1 activation by calcium /calmodulin), and various protein-protein interactions. Differences in the subcellular localization of the different PDEs are important for the functional compartmentalization of cAMP-mediated responses [28].

The interest in PDE1 in the pathogenesis of PKD arose from the facts that PC2 is a TRP channel with permeability to calcium and that PDE1 is the only PDE family directly regulated by calcium and calmodulin [29–32]. The PDE1 family consists of three members encoded by three different genes. PDE1A and PDE1B have a higher affinity for cGMP than for cAMP, while PDE1C is equally active for both. PDE1s integrate intracellular calcium levels and cAMP and cGMP signaling. Phosphorylation of PDE1A and PDE1C by PKA and of PDE1B by calcium/calmodulin-dependent protein kinase II reduces their activity via reduced affinity for calmodulin and sensitivity to calcium. PDE1 isoforms are dephosphorylated and reactivated by calcium/calmodulin-dependent protein phosphatase (calcineurin; protein phosphatase 2B).

PDE1 is the most abundant PDE in the distal nephron and collecting duct [33] and catabolizes a cAMP pool under the control of vasopressin and the vasopressin V2 receptor [16, 34]. Therefore we expected that the knockout of *Pde1a* would result in accumulation of cAMP, increased PKA-dependent phosphorylation and trafficking of AQP2 to the apical membrane of collecting duct principal cells, and enhanced urine concentrating capacity. Unexpectedly, however, we found that the *Pde1a* deficient mice were less able to concentrate the urine maximally and more capable to excrete an acute water load compared to wild- type controls. In retrospect, this could have been anticipated because phosphorylation and shuttling of AQP-2 to the plasma membrane and water permeability in the principal cells of collecting ducts is under the control of a compartmentalized pool of PDE4D bound to AKAP18delta in aquaporin-2 bearing vesicles [18]. Since PDE4 is activated by PKA-mediated phosphorylation, downregulation of PDE1 and subsequent activation of PKA may account for the increased PDE4 activity, reduced PKA-dependent phosphorylation of AQP-2, and concentrating defect observed in our study.

Interest in the role of cAMP and its interaction with calcium as regulators of cell proliferation dates back almost to its identification as the first known second intracellular messanger [35]. Remarkably, cAMP inhibits cell proliferation in some cells (e.g. vascular smooth muscle cells, mesangial cells, endothelial cells, fibroblasts, adipocytes, hepatocytes) while having the opposite effect in others (thyrocytes, pituitary cells, PC12 pheochromocytoma cells, granulosa cells, Sertoli cells, neurons, melanocytes) [36, 37]. The mechanisms by which cAMP modulates cell proliferation are complex, poorly understood and involve interactions with the RAS⁄RAF⁄mitogen-activated protein kinase (MAPK) and extracellular signal-regulated kinase (ERK) kinase (MEK)/ERK pathway at multiple levels [36, 37]. Cyclic AMP can stimulate or inhibit cell proliferation via activation or inhibition of ERK, respectively, as well as independently of this pathway, in a cell and context dependent manner. The type of response depends on the strength and duration of signaling, the subcellular localization of involved adenylyl cyclases, A-kinase anchoring proteins, PKA regulatory subunits, phosphodiesterases, and exchange proteins activated by cAMP (Epacs), and the relative expressions of Rap-1, B-Raf ver`sus Raf-1, and B-Raf isoforms, among others.

Previous studies have shown that cAMP stimulates proliferation of PKD-derived tubular epithelial cells while inhibiting proliferation of wild-type tubular epithelial cells [38–40]. These contrasting effects of cAMP are likely determined by the level of intracellular calcium, since the cAMP induced proliferation of ADPKD cells is reversed by activators of L-type calcium channels or by calcium ionophores. Similarly, the response of wild-type tubular epithelial cells to cAMP can be switched from inhibition to stimulation of proliferation by lowering free extracellular calcium or by calcium channel blockers. Inhibition of calcium dependent PI3K and Akt, which under normal calcium conditions phosphorylate and repress B-Raf, has been proposed to account for the proliferative effect of cAMP under conditions of calcium restriction.

The subcellular localization of the different PDEs is crucial for the functional compartmentalization of cAMP-mediated responses, and likely explains the lack of significant effect on total tissue cAMP and cGMP levels detected in PDE1A mutants despite a difference in PDE1 activity. Inhibitory and stimulatory effects of cAMP on cell proliferation have both been linked to a cAMP pool regulated by PDE3. In mesangial cells, PDE3 and PDE4 inhibitors increase cAMP levels and activate PKA to a similar extent, but only PDE3 inhibitors block phosphorylation of Raf-1 on serine 338, suppress Raf-1 kinase activity and ERK activation, and regulate proliferation [41, 42]. PDE3, but not PDE4 inhibitors, impede the proliferation of vascular smooth muscle and endothelial cells [43]. Despite the fact that PDE4 is three times more active than PDE3 in suspensions of renal cortical tubules, only PDE3 inhibitors suppress the proliferation of wild-type tubular epithelial cells following administration of folic acid [44]. On the other hand, only PDE3 inhibitors stimulate proliferation of MDCK cells (often used for cAMP inducible *in vitro* cystogenesis) despite the fact that PDE4 inhibitors are more effective in elevating intracellular cAMP levels in these cells [45].

The role of PDE1 in the control of cell proliferation has received less attention. In VSMCs, PDE1 inhibition or RNA interference of either PDE1A or PDE1C suppresses cell proliferation [46–48]. Since inhibition of PDE1 is expected to increase cGMP and cGMP inhibits PDE3, it is possible that downregulation of PDE1 indirectly affects a specific pool of intracellular cAMP under the control of PDE3. Interestingly, depletion of either, PDE1A or PDE3A using morpholinos causes pronephric cysts, body curvature, and hydrocephalus in zebrafish [12, 49]. A recent study has shown that inhibition of PDE1 or PDE3 stimulates the proliferation of ADPKD cells and that inhibition of PDE1 induces a mitogenic response to vasopressin in normal human kidney cells similar to the effect of restricting intracellular calcium [50]. These observations raise the possibility that PDE1 may function as a link connecting changes in intracellular calcium and the activity of a PDE3 pool controlling cell proliferation. The mild cystic disease observed in the present study affected the distal nephron (mainly the thick ascending limb of Henle) and collecting ducts, consistent with the pattern of expression of vasopressin V2 receptor [51, 52]. The knockout of *Pde1a* resulted only in a moderate reduction in PDE1 activity, likely due to redundancy with other PDE1 subfamilies, PDE1C and PDE1B.

We investigated whether *Pde1a* knockout mice had a cardiac phenotype for several reasons: i) Cardiovascular manifestations (hypertension, LV hypertrophy, cardiac valvular disease, cardiomyopathies, aortic root dilatation, arterial aneurysms and dissections, and pericardial effusion) are common causes of morbidity and mortality in ADPKD [22, 23]; ii) PDE1A has been proposed to play a role in the development of cardiac hypertrophy; iii) The polycystins are expressed in cardiac and arterial myocytes [53–56]; and iv) Knockdowns of either polycystin in mice and of PC2 in zebrafish impair myocardial function in the absence of renal cysts [57–60].

PDE1 is one of at least seven PDE families (PDE1 to PDE5, PDE8 and PDE9) present in mammalian hearts [61]. Reports on its importance relative to other PDE families and the relative expression of the three PDE1 subfamilies in different animal species have not been consistent (Table A in S1 File)[62–74]. Overall, our findings agree with some but not all published studies: i) PDE1 accounts for a large proportion of cAMP-PDE activity in the heart [65–67, 70]; ii) The protein expression of PDE1C is much higher than that of PDE1A in the myocardium [63, 64, 67, 72–74]; iii) Therefore, PDE1C is likely more important for cardiac hypertrophy than PDE1A; iv) The protein expression of PDE1A is much higher in adult aorta [32, 47, 75]; v) Consistent with this pattern of expression, *Pde1a* knockout mice in our study had lower blood pressures and a hyperdynamic circulation with an increased heart rate and ejection fraction. Interestingly, a large gene-centric meta-analysis in 87,736 individuals of European ancestry has identified an association between the *PDE1A* locus and diastolic and mean arterial pressures [76], and mutations of *PDE3A* resulting in increased PKA–mediated PDE3A phosphorylation and gain of function have been found in six families with hypertension and brachydactily syndrome [77]. On the other hand, PDE1 and PDE3 degrade cAMP in the juxtaglomerular cells thus inhibiting the release of renin and lowering blood pressure [78, 79].

While these observations do not support a role for a deficiency of PDE1A in the cardiac manifestations of ADPKD, a role for a defect in PDE1A, another PDE1 subfamily or another PDE family is still possible. In particular, it should be kept in mind that the consequences of inhibiting PDE1 on PDE3 activity may be different in a wild-type versus a PKD genetic background. While PDE1A is expressed at low level if at all in cardiac myocytes, it is highly expressed in the cardiac sinoatrial nodal cells where it regulates pacemaker function. Possibly, dysregulation of PDE1A activity could contribute to the increased prevalence of atrial fibrillation reported in ADPKD [80]. While PDE1C and PDE3A inhibitors attenuate cardiac hypertrophy and PDE3 inhibitors in the short term enhance LV contractility and overall systolic function, prolonged inhibition of PDE3 is detrimental because it promotes apoptosis and hypertrophy of the remaining cardiac myocytes [81].

In summary, PDE1A is strongly expressed in the kidney and the aorta. Knocking out *Pde1a* induced mild renal cystic disease and a urine concentrating defect (associated with upregulation of PDE4 activity and a decrease in protein kinase A dependent phosphorylation of aquaporin-2) on a wild-type genetic background and aggravated the renal cystic disease on a *Pkd2*^WS25/-^ background. *Pkd1a* mutants had lower aortic blood pressure and increased LV ejection fraction without a change in LV mass index, consistent with the high aortic and low cardiac expression of *Pde1a* in wild-type mice. These results support an important role of PDE1A in the renal pathogenesis of ADPKD and regulation of blood pressure.

## Supporting information

S1 File **Fig A.** Genomic DNA PCR amplification and PvuII-HF restriction endonuclease digestion of a 792 bp PCR product, showing a 669 and 123 bp fragments in the wild-type, a 777 bp fragment in Del15 and a 793 bp fragment in InsA (A, upper panel). With a longer run to better separate the upper bands, the lower 123bp band disappears (B, lower panel). **Fig B.** Axial and coronal MR images of Pde1a mutant mice at 6 and 12 months of age showing small renal cysts (arrows). Fi**g C**. Small cysts demonstrated by MRI in a 12 month-old, male wild type mouse (A) and by histology in 12 month old, female wild-type mouse (B)
(DOCX)
