# Supplementary material for: Generation and phenotypic characterization of Pde1a mutant mice
Source: PLoS One. 2017 Jul 27;12(7):e0181087. doi: 10.1371/journal.pone.0181087 (PMC5531505; doi:10.1371/journal.pone.0181087)
Supplement: S1 File — Fig A. Genomic DNA PCR amplification and PvuII-HF restriction endonuclease digestion of a 792 bp PCR product, showing a 669 and 123 bp fragments in the wild-type, a 777 bp fragment in Del15 and a 793 bp fragment in InsA (A, upper panel). With a longer run to better separate the upper bands, the lower 123bp band disappears (B, lower panel). Fig B. Axial and coronal MR images of Pde1a mutant mice at 6 and 12 months of age showing small renal cysts (arrows). Fig C. Small cysts demonstrated by MRI in a 12 month-old, male wild type mouse (A) and by histology in 12 month old, female wild-type mouse (B) (DOCX) [file pone.0181087.s001.docx]

**S1 File**

Methods

The Mayo Clinic Institutional Animal Care and Utilization Committee approved all experimental protocols for the work described within this report.

Targeted disruption of *Pde1a*. We used a TALEN pair to Pde1a exon 7 (NM_001159582.1 mouse chromosome 2). This exon was selected because it is the second exon in the catalytic domain and contains a histidine-aspartic acid dipeptide involved in the co-ordination of a Zn++ ion required for catalytic activity (1). The parent vector was pT3TS-GoldyTALEN donated to us by Dr. Stephen C. Ekker, Ph.D. (Mayo Clinic) and the TALEN array was assembled using `Golden Gate' assembly as described in Cermak et al(2). TALEN capped and polyadenylated RNA was generated using a mMACHINE T3 Transcription Kit. RNA was precipitated in lithium chloride washed in 70% ethanol and resuspended in injection buffer and checked for integrity by formaldehyde 0.8% agarose gel electrophoresis. RNA was injected at a concentration of 5ng/μl (vol=3-5pL) into the pronucleus of C57BL6/J zygotes. We injected a total of 100 zygotes which were transferred to pseudo pregnant outbred mice. We recovered 40 pups of which at least 5 males and 3 females harbored a mutation, of these we chose an inframe del15 and and a frame shifting A insertion.

Genotyping and breeding of *Pde1a* and *Pkd2* mutant mice. Tissue samples for genotyping were collected by tail clipping at 2 weeks of age into labeled microfuge tubes. Genomic DNA was extracted using the QIAamp DNA Mini Kit (Qiagen Inc., Valencia, CA). For *Pde1a* genotyping, the PCR product from forward primer GAACACACTAACGATGTCCC and reverse primer TGGTTTGAGTAACTGCCAC was digested with PvuII HF (Supplemental Figure 1). In the WT the uncut fragment is 792bp and upon restriction digestion with PvuII HF, this molecule is cut down to 669 bp which is visible on an agarose gel and 123bp which is barely visible. Both the *Pde1a*^InsA^ allele and *Pde1a^del15^* alleles have a disruption of the single PvuII HF target site and so never cut, hence the same assay can be used to genotype both *Pde1a* alleles (strains). In the case of the *Pde1a*^InsA^  allele this PvuII resistant fragment is 793bp and in the case of the *Pde1a^del15^* allele this fragment is 777bp. The 793bp and 777bp bands are indistinguishable on an agarose gel, resolving at c780bp. In short, when genotyping a *Pde1a*^InsA/+^ or a *Pde1a*^del15/+^ mouse, PCR and PvuII digestion results in a WT allele that runs at 669bp and both the mutant alleles run at a higher molecular mass of c780bp –- so that the high molecular weight band represents the mutant and the lower the WT. A WT has only a 669bp band, a heterozygote (*Pde1a*^InsA/+^ or a *Pde1a*^del15/+^ mouse) has a doublet of 669 and a c780bp and a homozygote mutant has a single band of c780bp.

Phenotypic characterization of *Pde1a* mutant mice. Litter sizes, blood and urine biochemistries and magnetic resonance imaging (MRI) of the abdomen and/or heart were obtained at different time points (6 and 12 months) in *Pde1a* mutant and wild-type mice. To examine whether the knockout of *Pde1a* affects the capacity to concentrate or dilute the urine, the urinary concentrating ability was tested after 24-h water deprivation and the capacity to excrete a water load was tested after intraperitoneal injection of 2 ml of sterile water at 12 months of age. Echocardiogram and aortic blood pressure and histology of *Pde1a*^Del15/Del15^ and *Pde1a*^InsA/InsA^ were compared to those of sex and age matched wild-type controls at 12 months of age.

Treatment with desmopressin. To determine whether the administration of a V2R agonist would affect the development of renal cystic disease on wild-type, *Pde1a* null or combined *Pde1a* null and *Pkd2* mutant genetic backgrounds, desmopressin (30 ng/100 g/hour) or saline vehicle was administered subcutaneously via osmotic minipump (Alzet 1004 replaced every 3 wk) to wild type and *Pde1a*^InsA/InsA^ mice, or to *Pde1a*^+/+^;*Pkd2*^-/WS25^ and *Pde1a*^Del15/Del15^;*Pkd2*^-/WS25^ mice between 4 and 16 weeks of age.

Abdominal MRI. Ultra high field (UHF) abdominal MRI images were acquired in a Bruker AVANCEIII-700 (16.4 T) vertical-bore MRI spectrometer, using a 38 mm volume RF coil as previously described (<https://www.jove.com/video/52757/use-ultra-high-field-mri-small-rodent-models-polycystic-kidney>). Scout images were collected using the three orthogonal planes (coronal, sagittal, and axial), to locate the kidneys and prescribe the geometry for imaging. The anatomical abdominal images were acquired using a turbo Rapid Acquisition with Relaxation Enhancement (RARE) sequence, 11-19 coronal slices with TR/TE 1500/9 ms, RARE factor 8, (matrix 256×256, FOV 2.56 × 2.56 cm, slice thickness 0.75 mm). Kidney volume was estimated from anatomical images using Analyze software system (Mayo Foundation, Biomedical Imaging Resource, Rochester, MN).

Cardiac MRI. UHF cardiac MRI images were acquired as previously described (<https://www.jove.com/video/52757/use-ultra-high-field-mri-small-rodent-models-polycystic-kidney>). Following scout images to determine the position and orientation of the heart, an Intra Gate-Fast Low-Angle Shot (ig-FLASH) sequence (TR/TE 3.5/1.45 ms, repetition 100, matrix 256×256, FOV 2.56 × 2.56 cm) was used to acquire the cardiac cine images. The images consisted of a stack of 1-mm-thick short axis contiguous slices, covering the left ventricle from base to apex. Image analysis was performed using Analyze software system (Mayo Foundation, Biomedical Imaging Resource, Rochester, MN). The end-diastole image was selected at each slice and the left ventricular epicardial and endocardial borders were carefully traced. The end-diastolic volume (EDV) was estimated by the sum of the ED area (endocardial border) at each image and multiplied by the slice thickness. The same procedure was repeated to calculate end-systolic volume (ESV) from the end-systolic images. The left ventricular volume (LVV) was calculated as the difference between endocardial and epicardial areas and multiplied by the slice thickness. Left ventricular mass (LVM) was estimated by multiplying LVV by the specific gravity of myocardium (1.05 g/ml).

Blood Collections and Tissue Harvesting. Mice were euthanized at 4 months or 12 months of age depending on the study. The animals were weighed and anesthetized with ketamine (60mg/kg) and xylazine (10mg/kg) intraperitoneally. Blood was obtained by cardiac puncture for determination of serum analytes. The right kidney and part of the liver were placed into pre-weighed vials containing 10% formaldehyde in phosphate buffer (pH7.4). These tissues were embedded in paraffin for histologic studies. The left kidney was immediately frozen in liquid nitrogen for determination of cAMP levels and PDE activities. Heart was divided in three parts, one part (apex) immediately frozen in liquid nitrogen for determination of cAMP levels and PDE activities, and the other two parts were for histology.

PDE Activities. Kidneys and hearts were homogenized in ice–cold homogenization buffer containing 50 mM Tris (pH 7.5), 0.25 M sucrose, 5 mM MgCl_2_, 1mM EDTA, 1mM EGTA, and 1mM dithiothreitol and supplemented with protease inhibitor tablets (Roche). PDE activities were measured using 1 mM cAMP as substrate in buffer containing 50 mM Tris (pH 7.5), 5 mM MgCl_2_, 4 mM 2-mercaptoethanol, and 0.1% BSA.(4) ^3^H-cAMP was included as a tracer for quantitation. PDE activity in aliquots incubated with 2 mmol EGTA without calcium and calmodulin was determined as basal activity. To determine the activity in a sample caused by a specific PDE, various activators or inhibitors were included in the assay. PDE3 and PDE4 activities were determined as cAMP-PDE activities inhibited by 10 μM cilostamide or rolipram, respectively. In aliquots to determine the activity of calcium -calmodulin– dependent PDE1, the reaction mixture also contained 2.01 mM CaCl_2_ to obtain 10 μM calcium and 10 μg/ml calmodulin. Total PDE activity was determined as the sum of the basal PDE and PDE1 activities. Assays were initiated by the addition of substrate and incubated for 10 minutes at 30°C. The reaction was stopped by incubation for 3 minutes at 100°C. Crotalus atrox snake venom was then added, and after a 15-minute incubation at 30°C, hydrolyzed nucleotides were separated using high–capacity preactivated ion exchange resin (FabGennix, Frisco, TX). Slurries were mixed thoroughly and left to stand for 15 minutes on ice before centrifugation at 12,000g for 3 minutes. The radioactivity in 150-μl aliquots of the resulting supernatants was determined by liquid scintillation counting. Hydrolysis of cAMP was linearly proportional to incubation time and enzyme protein. Specific activities were defined as picomoles of cAMP hydrolyzed per minute per 1 mg protein.

cAMP and cGMP Content. The kidneys were weighed and ground to fine powder under liquid nitrogen in a stainless steel mortar and homogenized in 10 volumes of 0.1M HCl. The protein concentration was measured by using BCA Protein Assay Kit (Pierce, IL). After centrifugation at 600g for 10 min, the supernatant may be further diluted in the 0.1M HCl and run directly in the assay or stored frozen for later analysis. The cAMP and cGMP contents were assessed by enzyme-linked immunosorbent assay, according to the manufacturer’s instructions (Enzo Life Sciences, Farmingdale, NY). Samples were taken in triplicate. The results were expressed in pmol/mg of protein.

Western blots. Kidney and heart lysate and cytosolic proteins, extracted using Pierce subcellular protein fractionation kit (Pierce cat#87790. Thermo Fisher Scientific Inc. Wyman, MA) according manufacture’s protocol, were used. Protein concentrations were determined with BCA protein assay kit (Pierce Chemical Co). Kidney protein was heated in a sample buffer, electrophoresed on SDS-polyacrylamide gel, and transferred to PVDF membrane. After blocking at room temperature for 1h and incubating with primary antibody overnight at 4^o^C, membranes were washed and incubated with secondary antibody for 1h at room temperature. Detection was performed using enhanced ECL (Pierce Chemical Co). Antibodies used were: PDE1A (12442-2-AP, Proteintech, Rosemount IL); PDE1B (ab14600, Abcam, Cambridge, MA); PDE1C (sc67323, Santa Cruz, CA); pSer269-AQP2 (ab110418, Abcam, CA). Membrane was stained using swift membrane stain kit (Geno Technology Inc. cat#786-677; St. Louis, MO) according to the manufacture’s protocol. Total protein stain was used as loading control, multiple proteins of a thin strip through the center of the lane running from top to bottom was used for quantification.(5)

RT-PCR of tissue RNA. Total RNA was extracted from mouse kidneys using RNeasy Plus Mini Kit (Qiagen 74134). 1μg of total RNA was reverse transcribed using SuperScript First-Strand Synthesis System (Invitrogen) in a total volume of 20μl at 37^o^C for 1hour to synthesize cDNA. The PCR reactions were performed with 200nM mouse *Pde1a* specific primers (Forward: 5’-ATGCAGCTGACGTCACTCAA, Reverse: 5’- AGGGCCATGGTCCATCTGTA) for 30 cycles at 95^o^C for 40s, 60^o^C for 1min and 72^o^C for 1min. 10μl of the PCR products were digested with 10μl of a digest master mix using 0.3μl of PvuII-HF per reaction and digested overnight. The PCR uncut product is 484 bp. PvuII cuts the PCR product to generate 327, 93, 58 and 6 bp fragments for wild-type and 420, 58, and 6 bp fragments for *Pde1a*^Del15^ and *Pde1a*^InsA^.

Histomorphometric analysis. Four-micrometer transverse tissue sections of the kidney, including cortex, medulla, and papilla, and the liver were stained with hematoxylin-eosin to measure cystic indices. Image analysis procedures were performed with Meta-Morph software (Universal Imaging, West Chester, PA). Digital images were acquired using a light microscope with a high–resolution Nikon Digital Camera (Nikon DXM1200). The observer interactively applied techniques of enhancement for a better definition of interested structures and to exclude fields too damaged to be analyzed. A colored threshold was applied at a level that separated cysts from noncystic tissue to calculate the cystic indices. Histomorphometric analyses were performed blindly without knowledge of group assignment.

Immunohistochemical analyses. Antibodies used were against lysozyme (ab108508, Abcam, Cambridge, MA), Tamm-Horsfall protein (THP, sc20631, Santa Cruz, CA), aquaporin-2 (AQP2, sc9882, Santa Cruz, CA), epithelial membrane antigen (EMA, MA5-11202, Thermo Scientific, Rockford, IL), cAMP–responsive binding protein (P-CREB, Cell Signaling Technology, Danvers, MA), and proliferating cell nuclear antigen (PCNA, sc-56, Santa Cruz, CA). To estimate proliferative indices fields (×400) of the renal cortex were randomly selected, and 1,000 tubular epithelial cell nuclei/tissue section were counted. Proliferative indexes were calculated as percentages of cells positive for PCNA.

Statistical analysis. Data are expressed as means ± SD. One-way analysis of variance (ANOVA) with post-hoc Tukey test is used for comparisons between groups. The Student’s *t*-test was used for comparisons between two groups.

References

1. Card GL, England BP, Suzuki Y, Fong D, Powell B, Lee B, et al. Structural basis for the activity of drugs that inhibit phosphodiesterases. Structure. 2004;12(12):2233-47.

2. Cermak T, Doyle EL, Christian M, Wang L, Zhang Y, Schmidt C, et al. Efficient design and assembly of custom TALEN and other TAL effector-based constructs for DNA targeting. Nucleic Acids Res. 2011;39(12):e82.

3. Wu G, D'Agati V, Cai Y, Markowitz G, Park J, Reynolds D, et al. Somatic inactivation of PKD2 results in polycystic kidney disease. Cell. 1998;93(2):177-88.

4. Deng C, Wang D, Bugaj-Gaweda B, De Vivo M. Assays for cyclic nucleotide-specific phosphodiesterases (PDEs) in the central nervous system (PDE1, PDE2, PDE4, and PDE10). Current Protocols in Neuroscience. 2007;Chapter 7:Unit 7 21.

5. Aldridge GM, Podrebarac DM, Greenough WT, Weiler IJ. The use of total protein stains as loading controls: an alternative to high-abundance single-protein controls in semi-quantitative immunoblotting. J Neurosci Methods. 2008;172(2):250-4.

**
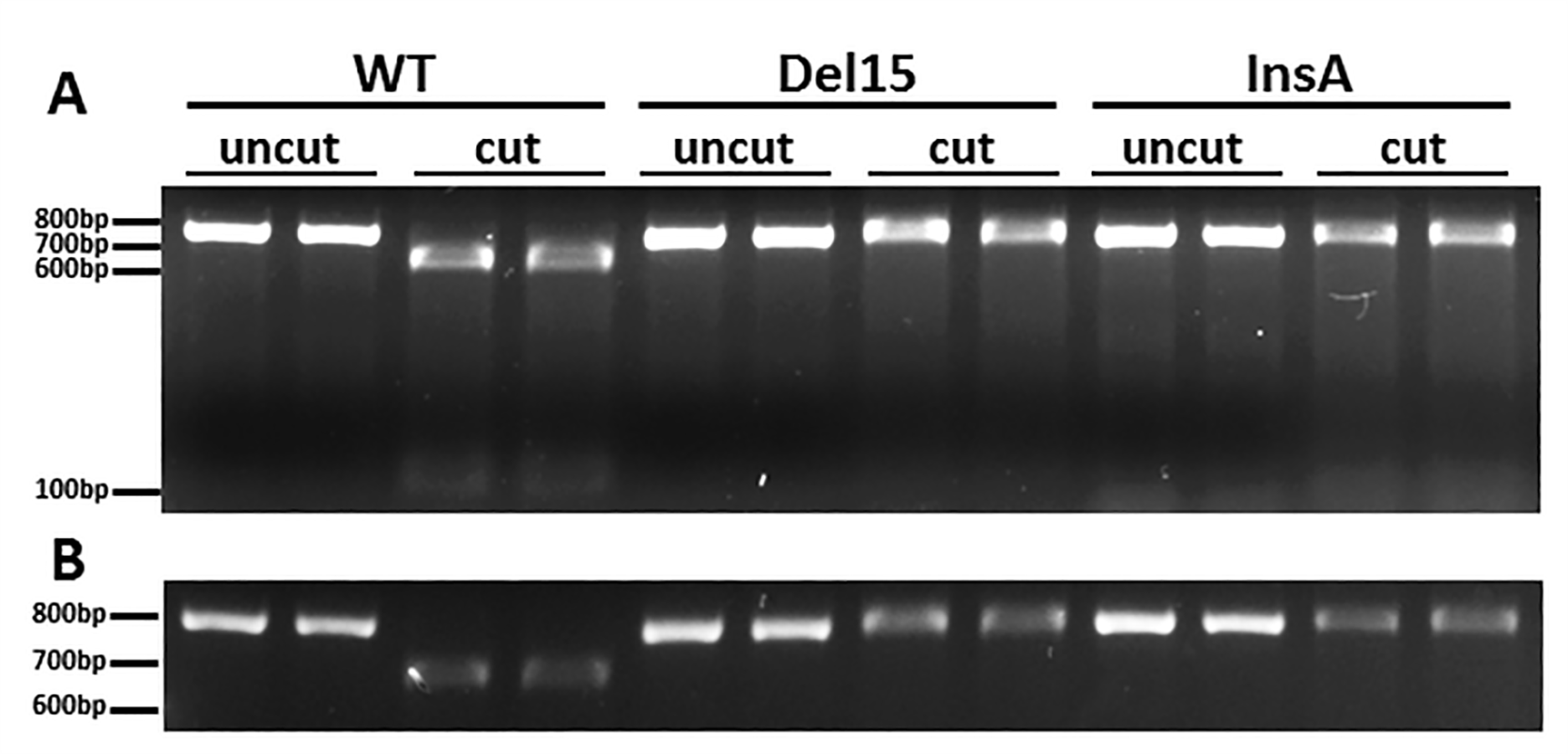
**

**Fig A.** Genomic DNA PCR amplification and PvuII-HF restriction endonuclease digestion of a 792 bp PCR product, showing a 669 and 123 bp fragments in the wild-type, a 777 bp fragment in Del15 and a 793 bp fragment in InsA ( A, upper panel). With a longer run to better separate the upper bands, the lower 123bp band disappears (B, lower panel).


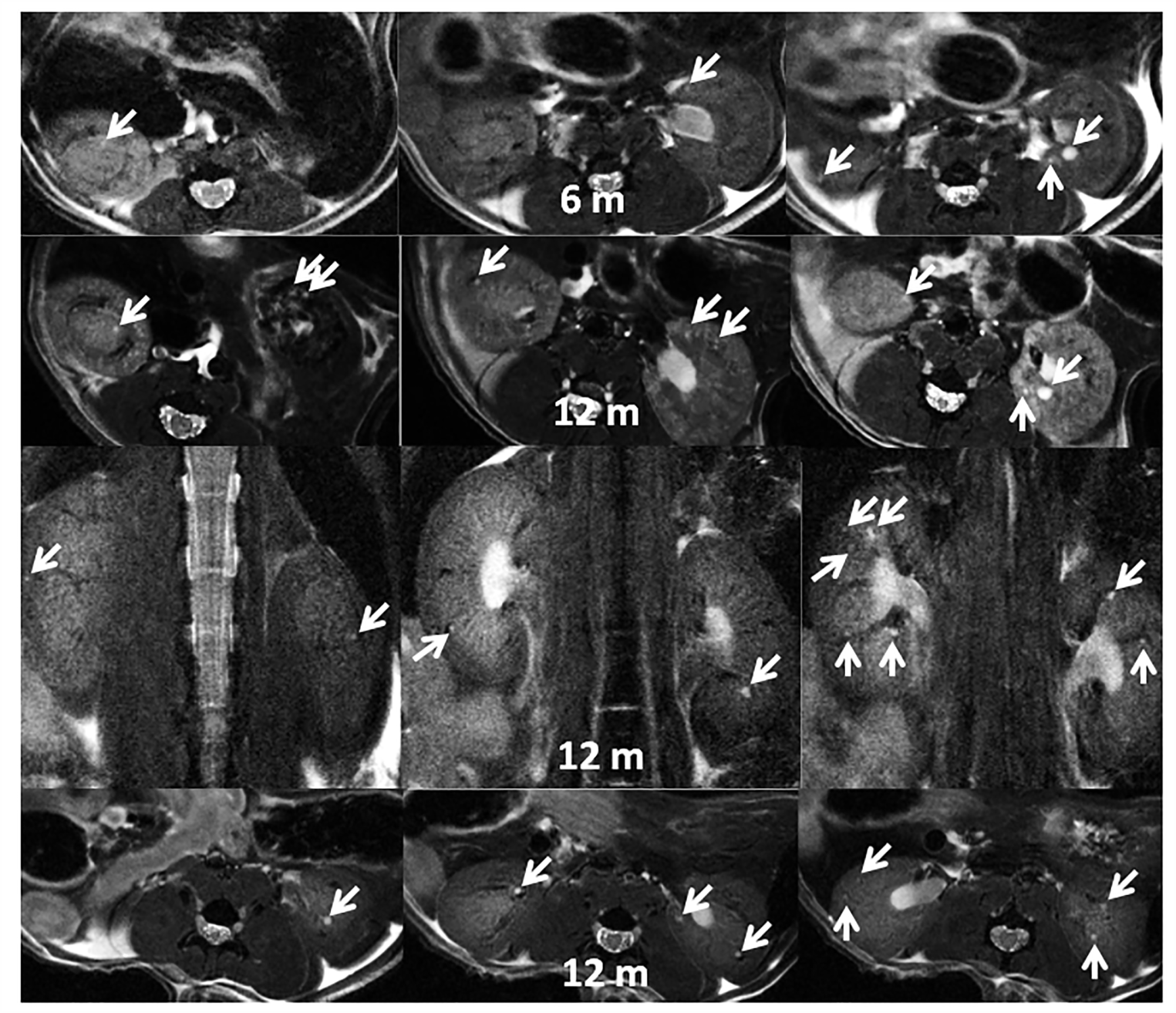


**Fig B.** Axial and coronal MR images of *Pde1a* mutant mice at 6 and 12 months of age showing small renal cysts (arrows).


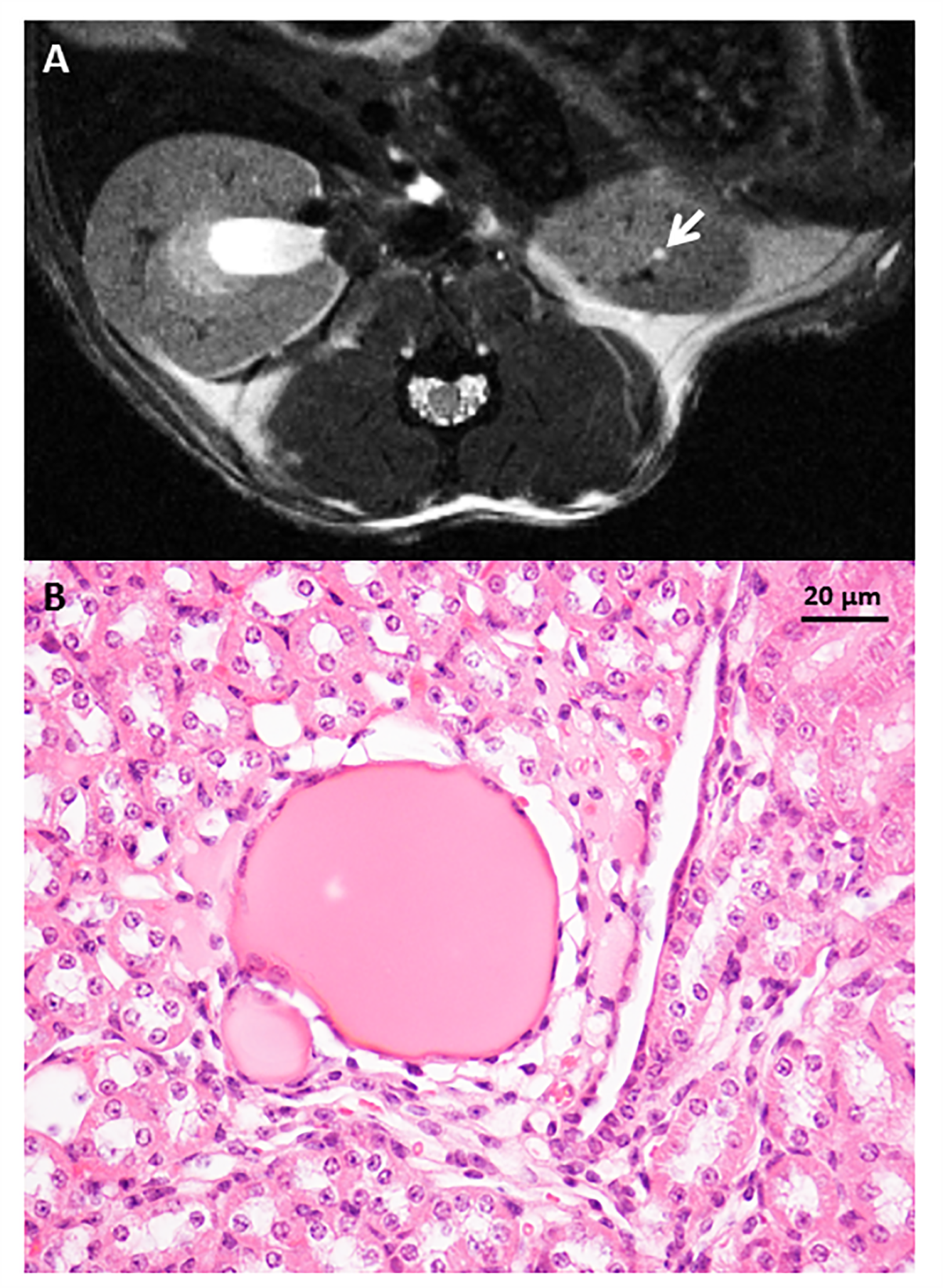


**Fig C** Small cysts demonstrated by MRI in a 12 month-old, male wild type mouse (A) and by histology in 12 month old, female wild-type mouse (B)

**Table A. Summary of reports on the cardiac expression of the PDE1 family.**

| Author, year, ref | Method | Species | Results |
| --- | --- | --- | --- |
| Bode, 1991 (59) | HPLC, IEC, activity | Rat ventricle | PDE1 activity restricted to non-myocyte elements |
| Kostic, 1998 (60) | RT-PCR | Rat heart | Only PDE1C is expressed |
| Sonnenburg, 1998 (61) | IHC | Rat heart | PDE1A restricted to arteries |
| Wallis, 1999 (62) | Activity | Human ventricle | PDE1 is the major PDE activity |
| Hambleton, 2005 (63) | Activity | Human ventricle | PDE1 is the major PDE activity |
| Vandeput, 2007 (64) | Western, IHC, activity | Human LV | PDE1C accounts for most cAMP PDE activity in soluble fractions and most cGMP PDE activity |
| Miller, 2009 (22) | RT-PCR | Human, rat, mouse ventricles | PDE1A mRNA and protein levels similar in the three. PDE1C protein lower in mouse and PDE1B not detectable |
|  | Treatment with Phenylephrine or Isoproterenol ± PDE1 inhibitor or PDE1A siRNA | Rat ventricular myocytes | PDE1A is upregulated in cardiac myocyte hypertrophy. PDE1 inhibitor or PDE1A siRNA prevents hypertrophy |
|  | Treatment with isoproterenol ± PDE1 inhibitor | Mouse | PDE1A is upregulated in cardiac hypertrophy. PDE1 inhibitor prevents hypertrophy. |
| Mokni, 2010 (65) | RT-PCR, activity | Rat left ventricle | PDE1 accounts for 7% and 16% of cAMP- and cGMP-PDE activities, respectively |
| Miller, 2011 (67) | RT-PCR, Western | Rat cardiac fibroblasts | PDE1A mRNA and protein are weakly expressed in resting fibroblasts |
|  | Treatment with Ang II ± PDE1A shRNA | Rat cardiac fibroblasts | Upregulation of PDE1A associated with myofibroblastic transformation and prevented by PDE1A shRNA |
|  | Treatment with isoproterenol ± PDE1 inhibitor | Mouse | Upregulation of PDE1A associated with cardiac fibrosis and prevented by PDE1 inhibitor |
| Johnson, 2012 (68) | RT-PCR, activity | Human, guinea pig, rat cardiac myocytes | PDE1C is the main PDE1 (low level in the rat) |
| Lakics, 2012 (66) | RT-PCR | Human heart | PDE1C is the main PDE1 |
| Knight, 2016 (23) | RT-PCR | Mouse cardiac myocytes and fibroblasts | PDE1C expressed in myocytes, negligible in fibroblasts |
|  | Treatment with Ang II ± isoproterenol | Mouse cardiac WT and PDE1C-KO myocytes | Upregulation of PDE1C associated with hypertrophy and cell death/apoptosis in WT but not in the PDE1C-KO myocytes |
|  | Transverse aortic constriction | WT and PDE1C-KO mice | Upregulation of PDE1C associated with myocardial hypertrophy and apoptosis, cardiac fibrosis and failure in WT but not in the PDE1C-KO mice |
| Lukyanenko, 2016 (69) | RT-PCR, Western, Activity | Rabbit LV myocytes and sinoatrial nodal cells (SANCs) | PDE1C much higher than PDE1A mRNA and protein in LV; the reverse in SANCs. PDE1 inhibitor reduced total PDE activity by 4% in LV, by 39% in SANC lysates. |
